# Senior physiotherapy students as standardised patients for junior students enhances self-efficacy and satisfaction in both junior and senior students

**DOI:** 10.1186/1472-6920-14-105

**Published:** 2014-05-23

**Authors:** Allison M Mandrusiak, Rosemary Isles, Angela T Chang, Nancy L Low Choy, Rowena Toppenberg, Donna McCook, Michelle D Smith, Karina O’Leary, Sandra G Brauer

**Affiliations:** 1 Division of Physiotherapy, School of Health & Rehabilitation Sciences, The University of Queensland, St Lucia, Brisbane QLD 4072, Australia; 2 Faculty of Health Sciences, Australian Catholic University Limited (McAuley Campus), Banyo, Brisbane QLD 4014, Australia

**Keywords:** Standardised patient, Peer-assisted learning, Physiotherapy

## Abstract

**Background:**

Standardised patients are used in medical education to expose students to clinical contexts and facilitate transition to clinical practice, and this approach is gaining momentum in physiotherapy programs. Expense and availability of trained standardised patients are factors limiting widespread adoption, and accessing clinical visits with real patients can be challenging. This study addressed these issues by engaging senior students as standardised patients for junior students. It evaluated how this approach impacted self-reported constructs of both the junior and senior students.

**Methods:**

Learning activities for undergraduate physiotherapy students were developed in five courses (Neurology, Cardiorespiratory and three Musculoskeletal courses) so that junior students (Year 2 and 3) could develop skills and confidence in patient interview, physical examination and patient management through their interaction with standardised patients played by senior students (Year 4). Surveys were administered before and after the interactions to record junior students’ self-reported confidence, communication, preparedness for clinic, and insight into their abilities; and senior students’ confidence and insight into what it is like to be a patient. Satisfaction regarding this learning approach was surveyed in both the junior and senior students.

**Results:**

A total of 253 students completed the surveys (mean 92.5% response rate). Across all courses, junior students reported a significant (all *P* < 0.037) improvement following the standardised patient interaction in their: preparedness for clinic, communication with clients, confidence with practical skills, and understanding of their strengths and weaknesses in relation to the learning activities. Senior students demonstrated a significant improvement in their confidence in providing feedback and insight into their own learning (*P* < 0.001). All students reported high satisfaction with this learning experience (mean score 8.5/10).

**Conclusion:**

This new approach to peer-assisted learning using senior students as standardised patients resulted in positive experiences for both junior and senior students across a variety of physiotherapy areas, activities, and stages within a physiotherapy program. These findings support the engagement of senior students as standardised patients to enhance learning within physiotherapy programs, and may have application across other disciplines to address challenges associated with accessing real patients via clinical visits or utilising actors as standardised patients.

## Background

Prior to transitioning into the clinical environment, it is optimal for health professional students to practise the integration and application of knowledge, skills and professional behaviours within a clinical context. Given that these students often find the transition to clinical environments highly stressful [1], it is important to provide exposure to clinical contexts within the study program and this is often addressed through clinical visits. However, there are known limitations to this approach as it is dependent on the ability of clinical facilities and educators to manage large numbers of students seeking a similar interactive patient experience, and can be demanding on clinician time. Growing student numbers coupled with changes in healthcare delivery mean that health professional students have increasingly limited access to patients, so novel methods of clinical teaching to prepare students for clinical practice need to be considered. This is paramount given that an underprepared student may pose safety risks to the patient, and places increased burden on the clinical educator [2].

One way to address the challenges associated with exposing students to clinical contexts has been to use standardised patients, who are individuals trained to present an illness or scenario in a systematic, unvarying manner [3]. The use of standardised patients permits students to learn and practice formative skills in a controlled and less threatening environment while allowing for provision of immediate feedback [4]. As such, standardised patients have been effective in developing health professional students’ clinical skills such as interpersonal communication [5] and patient interviewing and physical examination [3,6-9]. Use of standardised patients in pharmacy assisted in improving self-reported [10] and measured communication skills [5] and confidence [11], however this has not been investigated in physiotherapy students, who have a different set of needs in addition to communication skills including development of handling skills and ability to perform physical techniques.

One major disadvantage of using standardised patients is their expense. To address this, we developed an approach where senior (Year 4) physiotherapy students were trained to act in a standardised patient role for junior (Year 2 and 3) physiotherapy students. There are few reports of the impact of peer-assisted learning in which students act in a standardised patient role. Two studies have shown that there was no difference between interview communication skills in medical students who undertook training with their peers roleplaying as patients and those who interacted with trained standardised patients [12,13]. However, concerns have been raised that same year students acting as standardised patients may be too compliant in their responses to peers, may not be realistic, and may not be as thorough in giving feedback as trained standardised patients [12]. Although medical students reported reduced anxiety when same-year peers acted in a standardised patient role, it resulted in a less valuable experience compared with interactions with faculty as facilitators [14]. These concerns may be addressed by using senior students as standardised patients for junior students. Promisingly, Hudson and Tonkin [15] reported that when senior medical students (Year 6) facilitated junior students (Year 2) in a roleplaying task, there was no difference in learning outcomes compared with the gains made with faculty educators. The impact of engaging senior students as standardised patients on self-reported constructs such as confidence and insight into performance is not known and is important to optimise learning tasks.

In addition to considering the benefits of this approach for junior students, we explored the value of the experience for the senior student (standardised patient). There is a growing expectation for health professionals to develop the skills, attitudes and practices of competent teachers to be successful mentors. It is also well-recognised that learning is reinforced by the act of teaching other learners. Thus, this arrangement whereby senior students provide feedback to junior students through their role as a standardised patient may develop their own teaching, learning and mentoring skills, reinforce their discipline-specific knowledge and skills, and assist in the development of insight into the experiences of a patient. This is an important aspect of this learning experience to be investigated.

This study was therefore developed with two main aims: 1) to determine the impact of senior students acting as standardised patients on junior students’ (Year 2 and 3) self-reported confidence, preparation for clinic, communication, awareness of their own abilities, and satisfaction with the learning experience; and 2) to determine the impact of acting as a standardised patient on senior students’ (Year 4) self-reported confidence in providing feedback, awareness of what it is like to be a patient, understanding of peer-mentoring, and satisfaction with the learning experience. Secondary analyses investigated if there were any differences in results between junior years, or between courses.

## Methods

### Participants

Undergraduate physiotherapy students enrolled at The University of Queensland (Australia) in Year 2, 3, or 4 of their course were invited to participate in the evaluation of this learning experience. To be included, junior students had to provide written informed consent and to participate in the compulsory roleplaying session that was embedded in the course in which they were enrolled. Students were informed in advance about the nature of the evaluation related to the roleplaying sessions. If a student did not provide consent to complete the evaluation they were still expected to participate in the learning experience as part of the course. Senior students who were not on clinical placement at the time of the interactions were contacted by the year coordinator via email and invited to volunteer to participate. This study was approved by The University of Queensland Medical Research Ethics Committee.

### Procedure and setting

Learning activities that used a standardised patient format to address key client-centred activities were created by course coordinators. Standardised patient interactions were developed in five courses: two courses in Year 2 (Musculoskeletal A and B), and three courses in Year 3 (Musculoskeletal C, Neurology, and Cardiorespiratory). One activity was undertaken and evaluated in each of the three Musculoskeletal courses, two activities (i and ii) were undertaken and evaluated in the Neurology course, and one evaluation was undertaken following three activities in the Cardiorespiratory course. A course-specific standardised patient was developed for the senior students to portray. Groups of 5–6 junior students met with a standardised patient (senior student) to complete a patient interview and physical examination in a session ranging 1-2 hours. In Neurology ii and one of the three Cardiorespiratory activities, junior students undertook an aspect of patient management only (for example, mobilisation of a surgical patient) in a one hour session.

The standardised patient sessions took place in university practical teaching rooms and in the onsite Physiotherapy Musculoskeletal and Sports Injury Clinic. The senior students were provided with a script and details regarding how to play the case, and were coached in a one hour training session about their presentation for the interview and physical examination, as well as how to provide feedback to the junior students. All students stayed in role for the entire activity except during “time out” in which the scenario stopped and the students returned to their role as student rather than physiotherapist or patient and discussed aspects of the case and received or provided timely feedback, as well as at the end of the session during dedicated feedback time. Sessions were facilitated by physiotherapists experienced in education in the field who were briefed in their role in providing feedback and direction during the interaction.

### Survey

The 10 statements presented to the junior students immediately before and after the sessions are listed in Table 1 and cover communication, confidence, preparedness for clinic, and insight into their abilities. This was based on a survey from a previous study [11] around communication, expanded to investigate additional aspects including handling and practical skills. Three statements were given to the senior students which cover confidence in providing feedback, their understanding of what is required to be a peer mentor, and their awareness (insight) of what it is like to be a patient. Additional statements were presented to all students following the session, which asked about their satisfaction with the learning experience. Students rated their level of agreement with each statement by marking a visual analogue scale, anchored with zero (0) “strongly disagree” to 10 “strongly agree”.

**Table 1:** **Statements assessed by junior students pre and post the standardised patient interaction using a VAS**^**†**^

**Question**	**Statement**
1	I feel confident in my ability to complete this activity to a high standard.
2	I feel prepared for clinical placement in the area of physiotherapy associated with this role-playing activity.
3	I am aware of my strengths in this role playing activity.
4	I can identify areas of weakness related to this activity where I would benefit from further preparation for clinical placement.
5	I feel confident in my ability to establish rapport with a client.
6	I feel confident that I can use interpersonal skills such as reflective listening and appropriate use of questions when interacting with real clients.
7	I feel confident that I can provide information and education to clients.
8	I feel confident in my ability to use appropriate handling and practical skills with this client type.
9	I feel confident that I can interact in a professional manner.
10	I feel confident that I can identify key problems during an assessment.

^†^VAS = Visual Analogue Scale; 10 cm VAS anchored with ‘0’ (“Strongly disagree”) and ‘10’ (“Strongly agree”).

### Analysis

Paired t-tests were performed to determine if survey responses were different pre to post for each question in each course. Paired t-tests were performed to compare students’ responses across courses and independent t-tests were performed to compare across years. Descriptive statistics were undertaken to quantify post-session satisfaction responses. To determine if the role playing task influenced self-reported constructs, repeated-measures ANOVAs were undertaken on each year cohort, investigating the effect of training (pre, post), courses and question (1–10) and their interactions. The software package SPSS v17.0 (SPSS Inc., Chicago, IL, USA) was used for all analyses.

## Results

### Junior students

Data was collected from 202 junior students (see Table 2), which represented a mean response rate of 92% across all 6 surveys (range 87% to 95%).

**Table 2:** Characteristics of students by year cohort

	**Year 2**	**Year 3**	**Year 4**
*N*	101	101	51
Mean age in years (SD)	20.9 (3.03)	22.3 (5.1)	22.6 (2.8)
Gender: female (%)	60 (59.4%)	62 (61.4%)	34 (66.7%)

**Figure 1:**
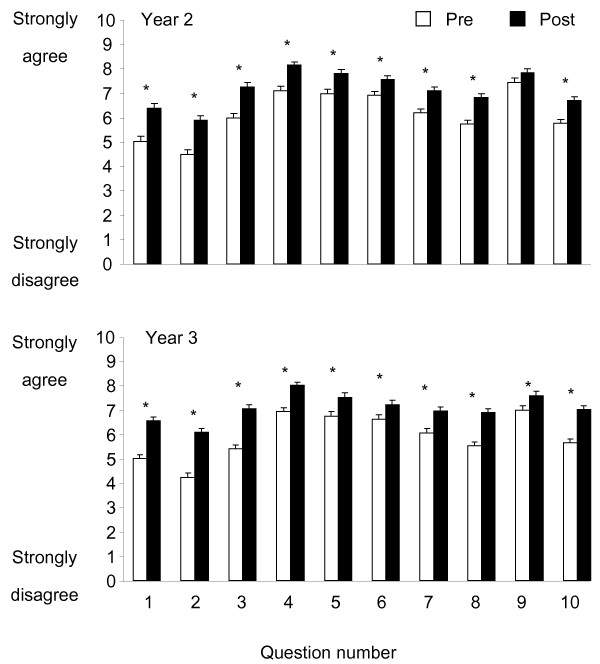
**Junior students self-reported constructs pre and post the standardised patient interaction.** Figure Legend: Mean ratings by junior students collapsed across courses in Year 2 and Year 3 for each of the 10 survey questions administered pre and post the standardised patient interaction. Error bars denote standard deviation.

In the Year 2 cohort, paired t-tests demonstrated that nine of the 10 questions showed a significant improvement from pre to post involvement in the standardised patient interaction (Figure 1, all *P* < 0.037). The question that did not show an improvement (*P* = 0.063) was Q9: “Confidence to act in a professional manner” as this question was rated highly both before (mean = 7.5/10) and after (mean = 7.8/10) the interaction. In this cohort, there was a significant training effect (*P* < 0.001) and question effect (*P* < 0.001) but no significant course effect (*P* = 0.124). There was no course x training interaction (*P* = 0.965), but significant interactions for course x question (*P* = 0.009), training x question (*P* < 0.001) and course x training x question (*P* < 0.001) combinations.

A similar overall result was found for the Year 3 cohort, for whom the paired t-tests demonstrated a significant improvement from pre to post involvement in the interaction for all questions, except Q9 for the Neurology i interaction which showed no difference between before (mean = 7/10) and after (mean = 7.3/10). When all Year 3 course interactions were combined, all ten questions showed a significant improvement (*P* < 0.001) from pre to post (Figure 1). Like the Year 2 students, there was a significant effect of training (*P* = 0.006) and question (*P* < 0.001), but also a course effect (*P* < 0.001). There was a training x question interaction (*P* < 0.001), but no further interactions (*P* > 0.075).

Following the session, junior students rated high satisfaction with this learning activity (mean = 8.6/10), felt they received helpful feedback from both senior students (mean = 8.5/10) and staff facilitators (mean = 8.9/10), felt it developed their problem-solving skills (mean = 7.9/10) and thought that the role-playing by the senior student was realistic (mean = 8.9/10).

### Comparison across courses

Responses were compared across the two Year 2 courses (Table 3). Before the session in Semester 1 (Musculoskeletal A), students scored themselves as more confident in their ability to undertake the activity (Q1) and more prepared for clinic (Q2) than before the session in Semester 2 (Musculoskeletal B). Similarly, following the activity they did not score themselves as highly in Semester 2 when providing information and education to clients (Q7) and identifying key problems in assessment (Q10).

**Table 3:** **Means (SD) of responses (scored on a 10 cm VAS**^**†**^**) pre and post the standardised patient interaction**

	**Year 2**				**Year 3**							
	**Musculoskeletal A**		**Musculoskeletal B**		**Musculoskeletal C**		**Neurology i**		**Neurology ii**		**Cardiorespiratory**	
**Question**	**Pre**	**Post**	**Pre**	**Post**	**Pre**	**Post**	**Pre**	**Post**	**Pre**	**Post**	**Pre**	**Post**
1.	5.5 (2.2)	6.5 (2.0)	4.6 (2.0)	6.3 (1.7)	4.0 (2.2)	6.0 (1.9)	5.3 (1.9)	6.7 (1.5)	5.2 (1.8)	6.5 (1.5)	5.4 (1.9)	7.0 (1.3)
2.	5.1 (2.3)	5.9 (2.4)	3.9 (2.1)	5.9 (1.8)	3.6 (2.3)	5.5 (2.1)	3.9 (2.0)	5.7 (2.0)	5.0 (1.9)	6.5 (1.7)	4.5 (2.2)	6.5 (1.4)
3.	6.2 (2.0)	7.2 (2.0)	5.8 (1.7)	7.3 (1.5)	5.0 (1.8)	6.7 (1.8)	5.5 (1.6)	6.7 (1.6)	5.7 (2.1)	7.1 (1.6)	5.5 (1.7)	7.6 (1.5)
4.	6.9 (2.2)	8.2 (1.9)	7.3 (1.7)	8.1 (1.2)	7.0 (2.1)	8.0 (1.3)	6.6 (1.9)	7.9 (1.2)	7.0 (2.0)	7.9 (1.1)	7.0 (1.8)	8.1 (1.4)
5.	6.8 (2.1)	7.9 (1.9)	7.2 (1.8)	7.8 (1.5)	6.7 (1.9)	7.4 (1.9)	6.5 (1.9)	7.3 (1.8)	6.9 (2.2)	7.6 (1.4)	6.9 (1.9)	7.7 (1.6)
6.	6.8 (2.0)	7.7(1.8)	7.0 (1.6)	7.4 (1.6)	6.5 (2.0)	7.2 (1.6)	6.5 (1.6)	6.9 (1.7)	6.8 (2.1)	7.3 (1.4)	6.7 (1.9)	7.4 (1.7)
7.	6.4 (1.9)	7.4 (1.9)	6.0 (1.8)	6.8 (1.5)	6.0 (2.2)	6.9 (1.8)	6.0 (1.8)	6.8 (1.5)	6.1 (1.9)	7.1 (1.5)	6.0 (1.8)	7.1 (1.5)
8.	5.9 (1.9)	7.0 (1.8)	5.5 (2.0)	6.7 (1.5)	5.3 (2.0)	6.5 (1.6)	5.4 (1.9)	6.8 (1.5)	5.4 (2.0)	7.0 (1.7)	5.8 (1.7)	7.2 (1.5)
9.	7.4 (2.2)	7.9 (2.1)	7.5 (1.8)	7.7 (1.5)	6.8 (2.2)	7.6 (1.7)	7.0 (1.6)	7.3 (1.8)	6.9 (2.1)	7.7 (1.2)	7.2 (1.7)	7.8 (1.6)
10.	6.1 (2.0)	7.1 (2.0)	5.6 (1.7)	6.3 (1.8)	5.1 (2.2)	6.9 (1.5)	5.5 (1.7)	6.8 (1.6)	6.0 (2.0)	7.0 (1.6)	5.8 (2.0)	7.3 (1.6)

^†^VAS = visual analogue scale; 10 cm VAS anchored with ‘0’ (“Strongly disagree”) and ‘10’ (“Strongly agree”).

Responses were compared across the three Year 3 courses (Table 3). Students reported the lowest confidence in their ability to undertake the activity (Q1) both before and after the Musculoskeletal C course (Semester 1) than the sessions in the other two courses (Neurology i and ii in Semester 1, and Cardiorespiratory in Semester 2). They scored their preparedness for clinic before and after the activity (Q2) lower in the first two sessions in Semester 1 (Musculoskeletal C and Neurology i) than the last two sessions (Neurology ii and Cardiorespiratory).

In the post-interaction survey, there were further differences in responses between courses, with students rating themselves more highly in the final interaction (Cardiorespiratory) than earlier courses, for questions regarding awareness of strengths (Q3), confidence in communication with clients (Q5, Q6, Q9) and preparedness for clinic (Q10).

### Comparison across years

Two similar session types were compared between courses in two year levels: Year 2 (Musculoskeletal A) and Year 3 (Musculoskeletal C). In both sessions, students undertook a patient interview and physical examination of a patient with a musculoskeletal injury in groups of 5–6 over 2 hours. Year 2 students rated their abilities before the session higher than Year 3 students in their confidence and preparedness for aspects of clinical practice (Q2, Q8 and Q10, all *P* < 0.049), awareness of strengths (Q3, *P* < 0.001), and confidence in the task (Q1, *P* < 0.001). Similarly, they rated themselves higher after the session in their ability to establish rapport with clients (Q5, *P* = 0.047).

### Senior students

Data was collected from 51 senior students who played the standardised patients, which represented a 93% response rate of the 55 who participated in the sessions (See Table 2). These students demonstrated a significant improvement in their responses to feeling confident about providing feedback (pre mean = 7.3/10, post mean = 8.6; *P* <0.001), having insight into what it is like to be a physiotherapy patient (pre mean = 6.9/10, post mean = 8.4; *P* <0.001), and an understanding of what it takes to be an effective peer-mentor (pre mean = 7.3/10, post mean = 8.3; *P* <0.001). Following the sessions, senior students rated the activity as being a useful learning experience for them (mean = 8.6/10) and for the junior students (mean = 8.9/10), and the activity helped them realise how much they had learnt (mean = 8/10).

## Discussion

This study is the first to investigate the impact of using senior physiotherapy students as standardised patients for junior students. It resulted in positive experiences for both the junior and senior students across a variety of courses, activities, and different stages within a physiotherapy program. This positive feedback about using standardised patients in a learning task is consistent with what has been reported in the literature [6,11], and may be due to several factors inherent to standardised patients including the use of a controlled and safe environment that may reduce anxiety compared with interactions with an actual patient, engagement in the experience and provision of timely feedback. It is likely these elements contributed to the high satisfaction with the learning experience, supporting the value of this model.

In the current study, junior students reported improvements in their confidence, communication with clients, practical skills, insight into their strengths and weaknesses related to this activity, and in their preparedness for clinical placement in this field of physiotherapy following their involvement in these standardised patient sessions. In general, these positive responses existed regardless of the area of study, the learning objective and the stage of learning across the program. This supports previous research that has shown improvements in self-reported communication [10] and confidence [11] in pharmacy students following engagement with trained standardised patients. The only area that did not show a significant improvement in some groups was confidence that they could interact in a professional manner. This is likely to be due to the high baseline score prior to participating in the activity that did not change after the interaction. Our findings support the use of experiential teaching with formative feedback, which is advocated to teach communication skills in physiotherapy [16].

Further, our results support a premise of junior students increasing their insight into their abilities across the program, and that the difficulty level of the task impacts perception of ability, such as feeling less confident in some aspects in the Semester 2 Musculoskeletal course than the equivalent Semester 1 course in the same year. Self-awareness and insight are cornerstone skills needed for the ability to reflect [17], where frequent and advanced reflection and self-assessment skills are features that distinguish experienced from novice physiotherapy clinicians [18]. It was pleasing that self-reported preparedness for clinic improved across the program.

Peer-teaching is a growing approach with established benefits in health professional education [19-21]. Senior students may provide similar advantages to peer educators in that junior students may feel more comfortable discussing performance issues with peers than with staff [22], and as the senior students have recently gone through the same experience may readily relate to the junior student and understand why the student is having difficulty [23]. Furthermore, senior students may have an advantage over peer educators in simulation sessions, as being in a different peer cohort may promote the development of professionalism. Similarly, using senior students in this role may address a number of the known challenges associated with accessing real patients via clinical visits or employing trained actors as standardised patients. Thus, the use of senior students is a novel and worthwhile approach to the standardised patient model.

The senior students found acting as a standardised patient to be a positive experience in terms of insight into what it is like to be a patient, their confidence in providing feedback, and their understanding of the mentoring process. Involvement as peer educators is reported to be beneficial to the students acting as educators by improving the acquisition and retention of knowledge and skills [24] and improving confidence [25,26]. Furthermore, it has been shown that medical students acting as peer tutors report a willingness to undertake further teacher training and to make teaching a major part of their professional practice [26]. This suggests that engagement in education may reinforce positive attitudes towards future teaching responsibilities, which is needed in a climate of increasing student numbers and one where the use of physiotherapy assistants is growing. These factors strongly support the need for physiotherapy graduates to understand the process of mentoring and their future professional teaching responsibilities, and these skills and insights may be developed through this model of standardised patients.

There are limitations to this research that should be addressed. Firstly, while there were over 200 participants in this study, all were from the one institution, and thus generalizability of results should be considered with caution. In addition, while the self-reported measures provide important information about perceived abilities and insights which are critical in building self-efficacy and reflective skills, it is important to understand the impact on actual ability or skill performance. This is an area for future research. This study pooled learning activities that varied in terms of skills developed or practiced (e.g. patient interview, physical examination, clinical reasoning, patient management) and number of interactions (single or multiple sessions within a course). It would be valuable to investigate these factors in more detail to help determine whether they may influence different learning objectives or stages of learning. Finally, this study did not aim to compare this type of learning experience with other modes, so it would be interesting to use a randomised trial to investigate the impact of senior students in these interactions compared with peers of the same year level or trained standardised patients, or with other types of learning activities.

## Conclusion

This study demonstrated that the use of senior students as standardised patients for junior students resulted in positive experiences for both junior and senior physiotherapy students, with significant improvements in reported self-efficacy and satisfaction across a range of discipline areas and year cohorts in a physiotherapy program. These findings support the engagement of senior students as standardised patients and may be an effective alternative to employing trained actors as standardised patients or accessing real patients where difficulties exist. Further investigation into this promising approach is indicated to optimise learning within entry level programs and enhance preparedness for clinical practice.

## Competing interests

The authors declare that they have no competing interests.

## Authors’ contributions

AM participated in collection of survey data, interpretation of results and development and revision of the manuscript. RI, AC, RT, NLC, DMcC, MS, KO’L participated in the design of the study, collection of survey data, interpretation of results, and drafting of the manuscript. SB participated in the design of the study, performed statistical analysis, interpretation of results, and drafting of the manuscript. All authors were involved in revising the manuscript for important intellectual content and have given final approval of the version to be published.

## Pre-publication history

The pre-publication history for this paper can be accessed here:

http://www.biomedcentral.com/1472-6920/14/105/prepub
